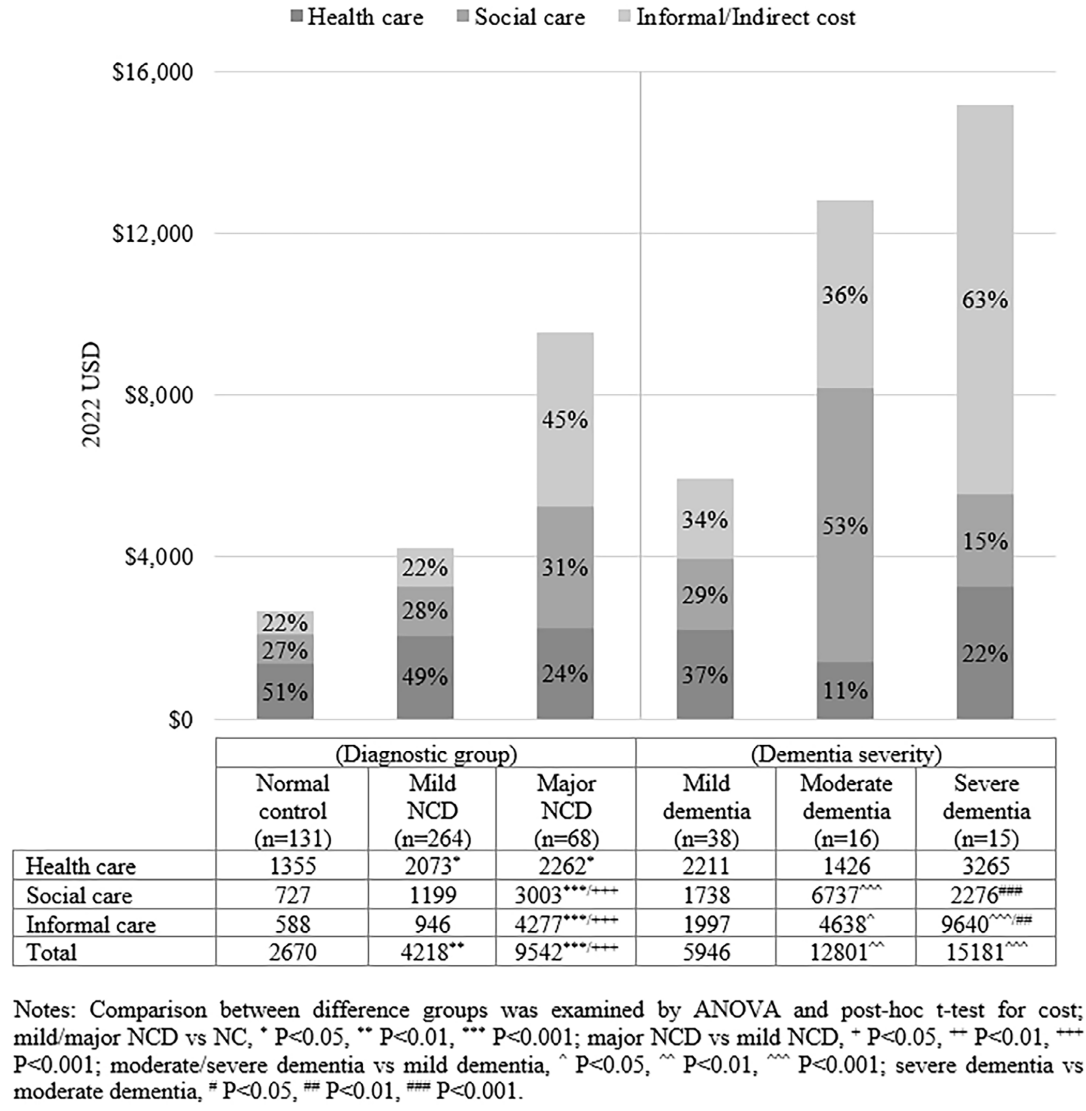# Healthcare utilisation and economic cost of neurocognitive disorders in Hong Kong community‐dwelling older adults: findings from a population‐based prevalence study

**DOI:** 10.1002/alz70860_102859

**Published:** 2025-12-23

**Authors:** Zhaohua Huo, Benjamin HK Yip, Allen Lee, Sheung Tak Cheng, Wai Chi Chan, Ada WT Fung, Suk Ling Ma, Calvin PW Cheng, Frank HY Lai, Samuel YS Wong, Linda Lam

**Affiliations:** ^1^ The Chinese University of Hong Kong, New Territories, Hong Kong SAR, China; ^2^ The Education University of Hong Kong, New Territories, Hong Kong SAR, China; ^3^ The University of Hong Kong, Hong Kong Island, Hong Kong SAR, China; ^4^ Hong Kong Baptist University, Kowloon, Hong Kong SAR, China; ^5^ Northumbria University, Newcastle, United Kingdom

## Abstract

**Background:**

Cost‐of‐illness studies are essential for care planning and policy making. It is increasing recognized that care for people living with neurocognitive disorders (NCDs) is costly. This study estimated the service utilisation and economic cost of mild and major NCDs among older adults in Hong Kong.

**Method:**

Based on a population‐based prevalence survey of NCDs in Hong Kong, a total of 461 community‐dwelling older adults aged 60 and over (major NCD: 68, mild NCD: 264, normal cognition: 129) completed the COI survey and were included in analysis. Service utilisation of participants, including medical, social, and informal care, was measured by the adapted version of Resource Utilisation of Dementia questionnaire. Per person cost was estimated from a societal perspective and expressed in 2022 US dollars. Associated factors of service utilisation and economic cost were examined by two‐part models fitting for mixed discrete‐continuous outcomes.

**Result:**

Annual cost per community‐living adult with major and mild NCD was US$9,542 (95%CI: 7,728‐11,356) and US$4,218 (3,670‐4,766) in Hong Kong, respectively, significantly higher than that of normal cognition. The territory‐wide cost of major and mild NCD in community was estimated at US$2.0 (1.7‐2.2) and US$1.5 (1.2‐1.8) billion, accordingly. The majority of cost of major NCD (76%) and mild NCD (50%) was attributable to social care and informal care. Costs of major NCDs even doubled from moderate to severe stages, and diagnosed cases incurred half and more costs than hidden cases.

**Conclusion:**

Economic burden of NCDs in Hong Kong is great, reflecting a striking social and care needs particularly in moderate and severe stages. Care planning should prepare for the blowout needs being revealed by hidden cases of NCDs, as well as the diversified needs by patients in different stages and their caregivers.